# Suitability of XRF for Routine Analysis of Multi-Elemental Composition: A Multi-Standard Verification

**DOI:** 10.3390/mps7040053

**Published:** 2024-07-05

**Authors:** Riccardo Fedeli, Luigi Antonello Di Lella, Stefano Loppi

**Affiliations:** 1 BioAgry Lab, Department of Life Sciences, University of Siena, 53100 Siena, Italy; 2 Department of Physical, Earth and Environmental Sciences, University of Siena, 53100 Siena, Italy; 3 NBFC—National Biodiversity Future Center, 90121 Palermo, Italy

**Keywords:** element quantification, microelement, macroelement, plant matrices, standard comparison, soil matrices, X-ray fluorescence

## Abstract

This study investigated the suitability of X-ray fluorescence (XRF) analysis for routine multi-elemental composition analysis, checking its analytical capabilities by measuring a wide array of certified reference materials of soil and plant origin. A portable XRF analyzer was used to evaluate 32 soil and 12 plant standard materials, using both the *Soil* and *Geochem* mode, with sequential beams, allowing the detection of a wide range of elements. Recovery rates were calculated by comparing XRF measurements with certified values, and their correlations were verified through the Spearman coefficient. The results demonstrated the reliability of XRF measurements for soil samples, with a large number of elements showing a good or very good recovery and strong correlations with certified values. For plant samples, XRF largely overestimated the certified values, but the strong statistically significant correlations for almost all tested elements allowed us to correct this systematic bias, using the reported median value for dividing the value obtained via XRF. The *Geochem* mode emerged as more reliable for a larger number of elements. It was concluded that XRF may be a suitable alternative to ICP-MS in routine multi-elemental composition analysis.

## 1. Introduction

Methods commonly and routinely used in multi-elemental composition analysis are ICP-OES (inductively coupled plasma–optical emission spectrometry) and ICP-MS (inductively coupled plasma-mass spectrometry). These methods are highly regarded for their accuracy, but are also criticized for being expensive, time-consuming, and prone to errors, especially due to the high degree of manipulation of the samples [1,2], since they require thorough solubilization of the investigated material; in addition, they can only be operated in controlled laboratory settings [3]. Instrumental neutron activation analysis (INAA) is a non-destructive technique that does not require pretreatment of samples, but the irradiation facilities to perform this kind of analysis are scarce and declining [4]. Particle-induced X-ray emission (PIXE) and X-ray fluorescence (XRF) are non-destructive techniques that also offer the advantage of having portable devices [5]. Both techniques are multi-elemental and can be used routinely for the determination of matrix composition of samples [6]. Comparison between these two latter techniques showed that they are largely complementary [7], but XRF is preferable, considering that the accuracy of PIXE is dependent on grain size and voids [8].

XRF devices are designed to be compact, durable, and energy-efficient, operating at various voltages and currents, thus being suitable for both laboratory and fieldwork [9]. The fundamental principle of XRF involves atoms in their ground state absorbing radiation at a specific frequency, becoming excited to a higher energy state, and then emitting fluorescence at characteristic wavelengths as light radiation [10]. This technique is used for quantitative analysis of the fluorescence intensity emitted by the vapor of the element being measured, under the excitation of radiation energy at a certain wavelength.

XRF provides a practical and cost-effective alternative to spectrometric ICP analysis, offering several advantages in various applications [11]. Firstly, portability and ease of use makes XRF particularly appealing for fieldwork. Portable XRF devices are compact and lightweight, allowing for on-site analysis without the need for sample collection and transportation to a laboratory [12]. This portability is especially advantageous in environmental monitoring, geological prospecting, and archeological excavations, where access to remote or difficult-to-reach locations is common. Additionally, XRF analyzers enable non-destructive analysis of samples, and this feature is invaluable when dealing with precious or irreplaceable materials, such as historical artifacts or valuable artworks, as well as biological materials. Unlike ICP-MS/ICP-OES, which requires sample destruction, XRF analysis preserves the integrity of the sample, allowing for further examination or testing, should it be necessary [13]. Speed is another significant advantage of portable XRF analyzers: with analysis times as rapid as a few seconds per sample, it is possible to obtain almost instantaneous results in the field. Real-time data acquisition is essential for applications that require immediate decision-making, such as collecting soil or plant samples with high levels of certain elements. Moreover, XRF analyzers are more cost-effective than ICP spectrometers: they have lower initial purchase costs and require less maintenance, making them accessible to a wider range of users and applications. This affordability makes portable XRF technology particularly attractive in academic research, small-scale laboratories, and resource-limited environments [14].

Despite this wide array of advantages, it is important to note that XRF devices have limitations, the most important being that they may not provide the same level of sensitivity or accuracy as ICP spectrometers for certain elements or at low concentrations.

This study aims to critically evaluate the accuracy and reliability of X-ray fluorescence analysis by directly comparing measurements obtained from a diverse and extensive array of certified reference materials. It is important to note the current limitations in the scientific literature, as there is a noticeable absence of comprehensive studies specifically designed to assess XRF’s suitability using such a wide range of certified standards, similar to those selected for this research (44 certified standards; 32 for soil matrices; and 12 for plant matrices). While the existing literature often compares XRF analysis with other techniques like ICP-MS or AAS, these comparisons typically do not encompass the breadth and specificity of certified standards used in this study.

By analyzing these certified reference materials, our research aims to establish a robust framework for evaluating the precision and accuracy of XRF quantifications across a spectrum of elements. This approach allows us to thoroughly investigate the measurement uncertainties associated with XRF, which are essential for ensuring reliable and reproducible results in routine analytical practices.

This study represents a significant contribution to advancing our understanding of XRF’s capabilities and limitations in delivering precise multi-elemental composition data. By addressing this research gap, we aim to provide valuable insights that enhance the credibility and applicability of XRF as a versatile analytical technique in various fields, including environmental science, geology, materials science, and industrial applications.

## 2. Materials and Methods

### 2.1. XRF Analyzer and Standard Analysis

A portable X-ray fluorescence analyzer (Olympus, Waltham, MA, USA) was used. This instrument (Vanta series C) features a Ag X-ray tube, operating within a voltage range of 15–40 kV, coupled with an integrated, large-area Silicon drift detector boasting a resolution of 165 eV. Vanta Desktop PC App *v.* 3.44 was employed in both *Soil* and *Geochem* modes, each employing three beams that operate sequentially, with acquisition times set at 20 sec per beam. This mode allows the detection of an extensive array of elements (Table 1). The certified limits of detection (LOD) of both modes are reported in Table 1. The LOD of portable XRF instruments in *Soil* and *Geochem* modes are determined by various factors including instrument calibration, sample composition, measurement time, and operating conditions, and they are reported in the materials provided by the company.

The *Soil* mode is optimized for the analysis of soils, sediments, and environmental materials. It is specifically calibrated to measure elements such as Lead (Pb), Arsenic (As), Cadmium (Cd), Mercury (Hg), and other heavy metals, which are often monitored to assess soil contamination. The calibration of this mode takes into account the typical matrices of soils and sediments, including moisture levels. This mode is commonly used in environmental applications, site remediation, and soil quality monitoring.

The *Geochem* mode, on the other hand, is optimized for the analysis of geological samples such as rocks, minerals, and drilling samples. It focuses on a wide spectrum of elements relevant to geochemistry, including Silicon (Si), Aluminum (Al), Calcium (Ca), Magnesium (Mg), Iron (Fe), and various rare earth elements. The calibration of the *Geochem* mode is specifically adapted to rocky matrices, with corrections for variable densities and the chemical complexities of geological samples. This mode is used in mineral exploration, core sample analysis, geochemical mapping, and geological studies.

Both modes offer high precision and accuracy for their respective fields of application but are optimized for different matrices. This means that a sample analyzed in a non-optimized mode may yield less accurate results. The detection ranges of the elements can vary between the two modes, as each is calibrated to maximize sensitivity for the specific elements of interest. Thirty-two soil certified reference materials and twelve plant certified reference materials, produced and certified by various accredited organizations and specialized research institutes (Table 2), were used for testing.

### 2.2. Recoveries and Statistical Analysis

Values for elements that were certified as below the instrumental LOD were disregarded from the statistical analysis, while values potentially detectable but measured as <LOD were included and recorded as the LOD value reported by the company. To determine the recovery rate from the certified standards, the values obtained through XRF were divided by the corresponding value of the certified standard. Recovery results are expressed as median ± uncertainty (%), the latter being defined as the 95% confidence interval of the median, calculated through 1000 iteration bootstrapping and divided by the median using the package “*simpleboot*” with the function “*one.boot*”. To evaluate the response of XRF analysis, correlations were tried between XRF values and certified values using Spearman’s coefficient. All statistical analyses were carried out using the R software v. 4.4.1 [15].

## 3. Results

### 3.1. Soil Matrices

The results obtained from the XRF analysis in both *Soil* and *Geochem* modes on the soil matrices are summarized in Table 3. Table S1 reported the mean, standard deviation, coefficient of variation, median, maximum, and minimum values for the recovery for both *Soil* and *Geochem* modes.

Recoveries calculated with uncertainty are classified based on their precision and the variability associated with the measurement. Here is how they are typically categorized: *Excellent*: A recovery is considered excellent if the measured value is very close to the expected value, with very low uncertainty. Typically, this means the measured value falls within ±10%. *Good*: A recovery is considered good if the measured value is close to the expected value, with moderate uncertainty. This could mean the measured value falls within ±20%. *Acceptable*: A recovery is considered acceptable if the measured value is reasonably close to the expected value, with a wider uncertainty range. This may include measured values within ±30%. *Not acceptable*: If the measured value is significantly far from the expected value, even with a wide uncertainty range, the recovery is considered not acceptable. *Very poor*: If the recovery is very low (e.g., less than 50%), even with associated uncertainty, it is considered very poor.

In the *Soil* mode, very good recoveries (±20%) emerged for Ca, Cu, K, Mg, Mn, Ni, Zn, Nb, Ni, Rb, Ti, and V; good recoveries (±30%) were found for Ba, Pb, and Zr; and recoveries in the range ± 40% emerged for Cr and Y. In the Geochem mode, very good recoveries (±20%) emerged for Al, Ca, Ce, Cr, Cu, Fe, Mg, Mn, Nb, Rb, Si, Ti, Zn, Zr, and Y; good recoveries (±30%) were found for Ba, Ni, Pb, and V; and recoveries in the range ± 40% emerged for Fe. Recoveries for Co, La, S, and Th were not satisfactory in both modes; interestingly, the recovery for Co was very low in *Soil* mode and very high in *Geochem* mode.

**Figure 1:**
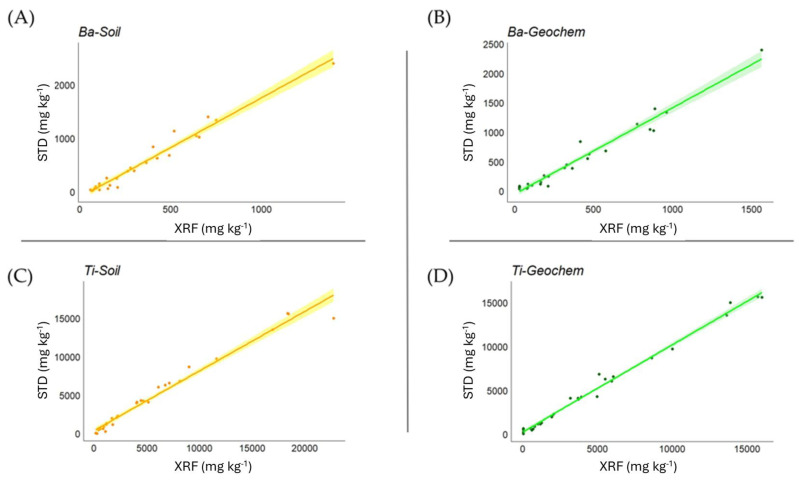
Relationship between measured and certified values for Ba and Ti in *Soil* (**A**,**C**) and *Geochem* (**B**,**D**) modes in soil matrices, expressed as mg kg^−1^.

Correlation analysis between soil certified values and XRF measurements (Table 4) overall showed a very high level of statistical significance. In the *Soil* mode, the strength of the correlation was very high (>0.9) for Ba, Ca, Cu, Fe, K, Mn, Ni, Pb, Rb, Ti, Zn, and Zr, while this was the case in the *Geochem* mode for Al, Ba, Ca, Fe, Mg, Mn, P, Pb, Rb, Si, Ti, Y, Zn, and Zr (good correlations in *Geochem* mode also emerged for Cu and Ni, 0.875 and 0.855, respectively). In Figure 1, examples are shown for Ba and Ti in both *Soil* and *Geochem* modes.

### 3.2. Plant Matrices

The results obtained from the XRF analysis in both *Soil* and *Geochem* modes on the plant matrices are summarized in Table 5. Table S2 reports the mean, standard deviation, coefficient of variation, median, maximum, and minimum values for the recovery for both *Soil* and *Geochem* modes. Only P and Pb in the *Soil* mode and Rb and Sr in the *Geochem* mode gave good or very good recoveries, while all the other elements showed very high recoveries.

**Figure 2:**
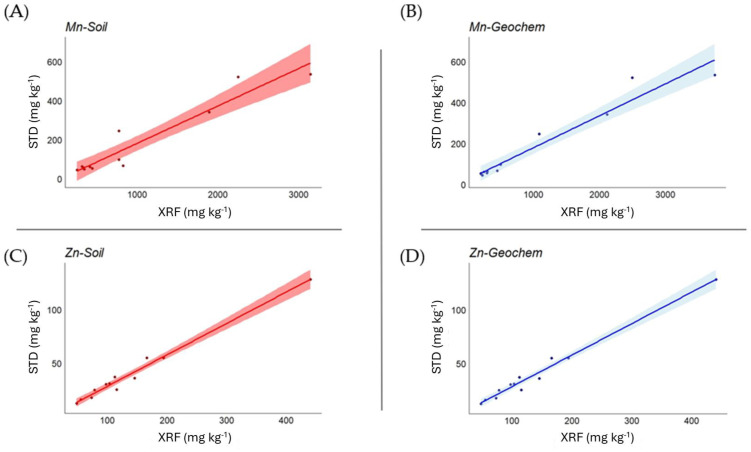
Relationship between measured and certified values for Mn and Zn in *Soil* (**A**,**C**) and *Geochem* (**B**,**D**) mode in plant matrices, expressed as mg kg^−1^.

Correlation analysis between plant certified values and XRF measurements (Table 6) showed, overall, a very high level of statistical significance. In the *Soil* mode, the strength of the correlation was very high (>0.9) for Cl, Cr, Fe, P, Pb, Sr, and Zn (good correlations emerged also for Ca, Cu, K, Mn, and S), while this occurred in the *Geochem* mode for Cl, Cu, Fe, Mn, Pb, Sr, and Zn (good correlations emerged also for Ca, P, Rb, and S). In Figure 2, examples are shown for Mn and Zn in both *Soil* and *Geochem* modes.

## 4. Discussion

Different research explores the correlations between mass ICP-MS and XRF analyses, for different types of matrices and fields of application.

Kilbride et al. [16] investigated the concentrations of As, Cd, Cu, Fe, Mn, Ni, Pb, and Zn in 81 soil samples using two types of field-portable XRF systems: a dual isotope system and an X-ray tube system. The metal concentrations obtained from the XRF systems were statistically compared with the results from the extractions followed by ICP analysis. A high degree of linearity was observed for Fe and Pb using the X-ray tube instrument and for Fe, Cu, Pb, Zn, Cd, and Mn with the dual isotope instrument. The performance of the XRF analyzers improved with longer analysis times for Cu, Mn, and Pb, whereas Fe, Zn, Cd, Ni, and As showed no significant improvement. Similarly, Li et al. [17] investigated the correlation between the concentrations of seven elements (As, Cd, Cu, Cr, Ni, Pb, and Zn) in one standard soil sample (GBW07403) and two other soil samples using XRF and ICP-MS. The results indicate that the concentrations of all elements meet the required precision standards for both methods. However, the relative error for Ni, As, Pb, and Cd was higher with XRF compared to ICP-MS. Regarding the two soil samples, concentrations were similar, except for Cd. Roullion and Taylor [18], Tian et al. [19], and Al-Maliki et al. [20] assessed the capability of XRF in environmental contamination research by comparing its performance in soil analysis with traditional laboratory methods. They found that XRF can provide comparable data to laboratory methods, especially for contaminants like Pb and As. Flemming et al. compared XRF with ICP-MS for analyzing trace elements in rice, concluding that XRF provides comparable performance to ICP-MS.

It is important to point out that the study did not report a comparison with certified standards and only measured the element concentration, comparing the results with ICP-MS. To the best of our knowledge, only four studies analyze certified standards, but none of these studies have scrutinized as many certified standards as our research has replicated. For instance, Barnett et al. [21] assessed the viability of XRF as an alternative to ICP measurements, albeit for only five elements (Co, Cr, Si, Ti, and Yb) in fecal material from sheep and cattle, finding a very good relationship between the two types of analysis. Similarly, Caporale et al. [22] delved into substituting XRF for ICP-MS quantification across various soil samples, concentrating on heavy metals. In their study, they conducted a quality assessment of the instrument using solely three certified standards: (*i*) ERM^®^ (European Reference Materials) CC141, (*ii*) ISE (International Soil Analytical Exchange) from the Wageningen Evaluating Programs for Analytical Laboratories, and (*iii*) NIST (National Institute of Standards & Technology). They reported recoveries in the range of 74–110% for the elements under investigation. Shand and Wendler [23] investigated the effectiveness of XRF in analyzing just seven certified soil standards, quantitatively assessing Ti, Cr, Mn, Fe, Ni, Cu, and As and qualitatively assessing Pb while also quantifying K, Ca, Zn, and Sr. Their analysis of ombrotrophic peat using XRF yielded satisfactory results for Cu (4.00 ± 1.00 mg/kg, certified 5.28 ± 1.04 mg/kg) and Pb (184 ± 3 mg/kg, certified 174 ± 8 mg/kg). However, XRF significantly overestimated the concentrations of Ca, Ti, Cr, Ni, and Zn by 2–3 times and Fe by 5 times compared to certified values. Roullion and Taylor [18] also investigated 11 certified soil samples. Elemental recoveries improved for all 11 elements post-calibration with reduced measurement variation and detection limits in most cases. The measurement repeatability of reference values ranged between 0.2 and 10% relative standard deviation, while the majority (82%) of reference recoveries were between 90 and 110%.In our study, the median values along with their respective uncertainties provide insights into the accuracy and reliability of the XRF measurements for the various elements investigated. Overall, our findings demonstrate the reliability of measurements conducted via XRF for soil samples, since a large number of elements had a good or very good degree of recovery and strong correlations with certified values. For plant samples, XRF largely overestimated the certified values, but in light of the strong statistically significant correlations (*r* > 0.800) for almost all tested elements, it is easily feasible to correct this systematic bias, by simply dividing the XRF value obtained by the respective median value reported in Table 5. It is important to note that the correlations observed for soil and plant samples are notably robust, in consideration of the large number of certified standards examined. Lastly, our findings showed that the *Geochem* mode provides reliable results for a larger number of elements.

To the best of our knowledge, our study represents a pioneering effort as the first to report results using plant matrix standards and analyze an unprecedented number of certified standards (44 total; 32 for soil matrices and 12 for plant matrices). This groundbreaking analysis not only expands the scope of portable XRF application but also enhances confidence in its reliability and precision for elemental analysis of both soil and plant samples.

In summary, our research significantly advances the understanding and utilization of XRF technology in elemental analysis. It establishes XRF as a tool capable of providing reasonably accurate and consistent results across diverse sample types and analytical environments. These findings underscore the importance of integrating XRF into scientific research where elemental characterization is essential, thereby solidifying its role in advancing analytical capabilities.

## Figures and Tables

**Table 1:** Limits of detection (LOD) of the two modes (*Soil* and *Geochem*) of the XRF expressed as mg kg^−1^.

	*Soil*	*Geochem*
Ag	4	2
Al	-	480
As	1	3
Au	-	8
Bi	5	4
Ca	30	40
Cd	5	3
Cl	40	30
Co	3	20
Cr	20	250
Cu	3	5
Fe	4	7
Hg	2	2
K	20	40
Mg	-	5030
Mn	10	15
Mo	2	3
Nb	1	1
Ni	4	5
P	250	30
Pb	2	4
Rb	1	2
S	40	30
Sb	8	4
Se	1	4
Si	-	320
Sn	7	3
Sr	2	2
Th	4	3
Ti	20	30
U	4	5
V	10	260
W	4	1
Y	2	2
Zn	2	20
Zr	2	1

**Table 2:** Certified reference materials used.

Standard	
*Soil*	*Plant*
ACE	B.C.R.-1135
AGV-2	GBW07603
ALI	GBW07604
ANG	IAEA-336
BCR2	IAEA-392
BEN	M1
BR	M2
DRN	M3
DTS-1	NFA-36
GA	NIST-1515
GBW07108	NIST-1543
GBW07311	NIST-1573a
GBW07411	
GH	
GS-N	
GSP-2	
JB-3	
JGb-1	
JR1	
JR-2	
JSO-2	
MAN	
MICA-FE	
NIM-G	
NIM-N	
NIM-P	
NIM-S	
NIST-1944	
SDC-1	
SDO-1	
SY-3	
UB-N	

**Table 3:** Recoveries (median ± uncertainty, where just the uncertainty is expressed as %) obtained using the *Soil* and *Geochem* modes on the soil matrices.

	*Soil*	*Geochem*
Al	-	1.08 (5.5)
Ba	0.71 (24.5)	0.78 (11.1)
Ca	1.06 (8.2)	0.89 (6.0)
Ce	-	0.82 (99.8)
Co	0.08 (87.6)	5.63 (75.2)
Cr	0.65 (16.8)	0.86 (32.5)
Cu	0.98 (15.1)	1.13 (19.7)
Fe	-	0.90 (2.5)
K	1.16 (10.5)	-
La	0.55 (46.9)	1.69 (177.1)
Mg	-	1.16 (12.4)
Mn	0.90 (7.8)	0.89 (3.5)
Nb	0.80 (15.3)	0.86 (9.5)
Ni	1.00 (11.3)	0.71 (33.6)
P	0.47 (42.8)	1.37 (26.2)
Pb	0.73 (13.6)	0.72 (11.6)
Rb	0.88 (2.9)	0.89 (2.5)
S	5.40 (40.6)	3.93 (62.1)
Si	-	1.02 (2.4)
Th	0.38 (62.1)	0.42 (56.2)
Ti	1.05 (9.4)	0.93 (6.1)
U	-	0.57 (46.7)
V	1.14 (68.3)	0.77 (35.1)
Y	0.66 (17.3)	0.90 (6.7)
Zn	0.97 (4.8)	1.03 (6.8)
Zr	0.72 (5.9)	0.81 (7.9)

**Table 4:** Spearman correlation coefficient of the element using both XRF’s mode (*Soil* and *Geochem*) on the soil matrices. ** *p* value < 0.01; *** *p* value < 0.001.

	*Soil*	*Geochem*
Al	-	0.934 ***
Ba	0.946 ***	0.965 ***
Ca	0.994 ***	0.961 ***
Ce	-	0.45
Co	0.333	0.700 ***
Cr	0.664 ***	0.656 ***
Cu	0.916 ***	0.855 ***
Fe	0.994 ***	0.994 ***
K	0.975 ***	-
La	0.448	0.363
Mg	-	0.923 ***
Mn	0.976 ***	0.984 ***
Nb	0.640 ***	0.646 ***
Ni	0.933 ***	0.875 ***
P	0.800 ***	0.900 ***
Pb	0.951 ***	0.949 ***
Rb	0.940 ***	0.932 ***
S	0.560 **	0.298
Si	-	0.920 ***
Th	0.836 ***	0.676 **
Ti	0.983 ***	0.981 ***
U	-	0.744 ***
V	0.673 ***	0.729 ***
Y	0.754 ***	0.966 ***
Zn	0.995 ***	0.993 ***
Zr	0.988 ***	0.989 ***

**Table 5:** Recoveries (median ± uncertainty, where just the uncertainty is expressed as %) obtained using the *Soil* and *Geochem* modes on the plant matrices.

	*Soil*	*Geochem*
Al	-	8.24 (131.8)
Ba	3.79 (95.6)	-
Ca	2.52 (64.8)	4.87 (85.8)
Cl	3.28 (79.7)	7.80 (147.4)
Cu	5.15 (34.8)	4.23 (24.2)
Fe	3.63 (23.5)	4.29 (18.9)
K	3.26 (35.5)	-
Mn	5.86 (20.7)	4.89 (13.3)
P	0.99 (26.5)	2.96 (37.6)
Pb	0.90 (66.9)	2.85 (63.6)
Rb	1.44 (86.5)	1.15 (22.6)
S	2.44 (51.3)	4.45 (81.5)
Sr	2.95 (109.8)	1.23 (63.9)
Zn	3.43 (9.7)	3.88 (8.8)

**Table 6:** Spearman correlation coefficient of the element using both XRF’s mode (*Soil* and *Geochem*) on the plant matrices. * *p* value < 0.05; ** *p* value < 0.01; and *** *p* value < 0.001.

	*Soil*	*Geochem*
Al	-	−0.429
Ba	0.512	-
Ca	0.794 ***	0.733 *
Cl	0.952 ***	0.988 ***
Cu	0.800 **	0.929 ***
Fe	0.916 ***	0.916 ***
K	0.879 **	-
Mn	0.867 ***	0.965 ***
P	0.915 ***	0.818 **
Pb	0.900 *	0.900 *
Rb	0.690 ***	0.857 ***
S	0.750 ***	0.750 ***
Sr	0.975 **	0.975 **
Zn	0.942 ***	0.979 ***

## Data Availability

Data are available on reasonable request from the corresponding author.

## Supplementary Materials

The following supporting information can be downloaded at: https://www.mdpi.com/article/10.3390/mps7040053/s1, Table S1: Mean, standard deviation, coefficient of variation, median, maximum, and minimum values for the recovery for both *Soil* and *Geochem* modes for soil matrices; Table S2: Mean, standard deviation, coefficient of variation, median, maximum, and minimum values for the recovery for both *Soil* and *Geochem* modes for plant matrices.
