# Supplementary material for: Suitability of XRF for Routine Analysis of Multi-Elemental Composition: A Multi-Standard Verification
Source: Methods Protoc. 2024 Jul 5;7(4):53. doi: 10.3390/mps7040053 (PMC11270293; doi:10.3390/mps7040053)
Supplement: Supplementary file 1 [file mps-07-00053-s001.zip › mps-3065487-supplementary.pdf]

Table S1. Mean, standard deviation (SD), coefficient of variation (CV), median, maximum, and minimum values for the recovery for soil matrices in both *Soil* and *Geochem* modes.

	Soil						***Geochem***					
	Mean	*SD*	*CV*	*Median*	*Max*	*Min*	*Mean*	*SD*	*CV*	*Median*	*Max*	*Min*
**Al**	-	-	-	-	-	-	1.21	0.82	67.58	1.08	5.64	0.76
**Ba**	1.04	0.70	67.18	0.71	2.72	0.46	0.90	0.43	48.26	0.78	2.43	0.35
**Ca**	1.10	0.17	15.81	1.06	1.43	0.82	1.03	0.39	37.90	0.89	2.44	0.52
**Ce**	-	-	-	-	-	-	1.32	1.03	78.31	0.81	3.33	0.06
**Co**	0.18	0.23	127.80	0.08	0.97	0.01	7.61	7.15	93.92	5.63	25.33	0.12
**Cr**	1.58	4.29	270.81	0.65	23.45	0.05	2.55	6.16	241.12	0.87	30.33	0.17
**Cu**	1.65	1.82	110.34	0.98	8.57	0.14	1.60	1.86	116.02	1.13	9.40	0.25
**Fe**	1.01	0.27	26.94	1.00	2.02	0.67	0.91	0.11	11.52	0.90	1.41	0.74
**K**	1.45	1.13	77.86	1.16	6.73	0.80	-	-	-	-	-	-
**La**	0.55	0.28	49.71	0.55	1.00	0.15	3.77	3.94	104.69	1.69	12.20	0.61
**Mg**	-	-	-	-	-	-	1.35	0.73	54.09	1.16	3.58	0.46
**Mn**	0.92	0.28	30.45	0.90	1.83	0.10	0.94	0.25	26.81	0.89	2.07	0.68
**Nb**	1.30	1.45	111.38	0.80	7.14	0.00	1.08	1.53	141.34	0.86	8.57	0.00
**Ni**	1.41	1.50	106.16	1.00	8.00	0.38	0.78	0.70	89.32	0.71	3.73	0.14
**P**	0.67	0.69	102.51	0.47	3.32	0.13	3.58	5.94	166.05	1.37	30.93	0.95
**Pb**	0.76	0.51	66.99	0.73	2.57	0.08	0.86	0.62	72.28	0.73	3.50	0.23
**Rb**	0.89	0.28	31.77	0.88	2.00	0.03	0.86	0.30	35.12	0.90	1.75	0.03
**S**	31.06	58.51	188.39	5.40	211.25	1.11	25.27	53.62	212.21	3.93	184.33	0.15
**Si**	-	-	-	-	-	-	1.01	0.11	10.53	1.02	1.23	0.61
**Th**	1.81	6.30	347.38	0.38	28.57	0.06	0.65	0.80	123.74	0.42	3.71	0.10
**Ti**	1.49	1.54	103.48	1.05	9.17	0.68	0.81	0.33	41.15	0.92	1.27	0.05
**U**	-	-	-	-	-	-	0.53	0.28	52.85	0.57	0.93	0.11
**V**	9.10	32.00	351.82	1.14	170.00	0.01	0.72	0.37	50.97	0.77	1.35	0.17
**Y**	0.71	0.45	63.27	0.66	2.00	0.02	0.95	0.31	33.12	0.90	2.00	0.10
**Zn**	1.03	0.26	24.79	0.97	2.00	0.75	1.12	0.31	28.07	1.03	2.33	0.75
**Zr**	0.75	0.27	36.65	0.72	2.00	0.30	0.85	0.23	26.61	0.82	1.75	0.43

Table S2. Mean, standard deviation (SD), coefficient of variation (CV), median, maximum, and minimum values for the recovery for plant matrices in both *Soil* and *Geochem* modes.

	Soil						***Geochem***					
	Mean	*SD*	*CV*	*Median*	*Max*	*Min*	*Mean*	*SD*	*CV*	*Median*	*Max*	*Min*
**Al**	-	-	-	-	-	-	13.46	10.96	81.44	8.24	33.05	3.21
**Ba**	5.49	6.51	118.59	3.79	18.61	0.06	-	-	-	-	-	-
**Ca**	2.88	1.20	41.70	2.52	5.64	1.70	6.97	4.16	59.73	4.88	14.96	3.14
**Cl**	5.84	5.34	91.39	3.29	17.09	1.75	16.36	16.21	99.07	7.81	50.74	6.00
**Cu**	6.04	2.82	46.69	5.16	11.97	3.20	4.41	2.31	52.35	4.23	9.31	0.89
**Fe**	4.05	1.30	32.07	3.63	6.77	2.46	4.74	1.18	24.95	4.30	7.01	3.35
**K**	3.88	1.43	36.79	3.26	6.65	2.30	-	-	-	-	-	-
**Mn**	6.42	2.29	35.70	5.86	12.16	3.13	5.11	1.02	20.04	4.90	7.00	3.78
**P**	1.02	0.31	29.98	0.99	1.57	0.53	3.15	1.25	39.59	2.97	5.22	1.04
**Pb**	0.96	0.45	46.42	0.90	1.53	0.47	2.97	1.34	44.93	2.86	4.80	1.68
**Rb**	3.01	4.73	157.22	1.44	14.71	0.74	1.09	0.36	33.31	1.15	1.54	0.44
**S**	3.06	1.24	40.54	2.45	5.47	1.96	5.61	2.91	51.86	4.45	10.27	2.90
**Sr**	3.36	3.91	116.24	2.95	9.98	0.22	1.10	0.70	63.50	1.23	2.07	0.22
**Zn**	3.55	0.47	13.39	3.44	4.53	3.02	3.82	0.39	10.27	3.89	4.45	3.19
